# Activities of asymmetric dimethylarginine-related enzymes in white adipose tissue are associated with circulating lipid biomarkers

**DOI:** 10.1186/1758-5996-4-17

**Published:** 2012-04-30

**Authors:** Hiroaki Iwasaki

**Affiliations:** 1 Department of Internal Medicine, Division of Endocrinology and Metabolism, Toshiba Rinkan Hospital, 7-9-1 Kami-tsuruma, Minami-ku, Sagamihara, Kanagawa, 252-0385, Japan; 2 HAR Research Institute, Misawa 4-13, Hino, Tokyo, 191-0032, Japan

**Keywords:** Protein *N*-arginine methyltransferase 1, Dimethylarginine dimethylaminohydrolase 1 and 2, Non-esterified fatty acids, Triglycerides, Type 2 diabetes mellitus

## Abstract

**Background:**

Asymmetric *N*^G^,*N*^G^-dimethylarginine (ADMA), an endogenous inhibitor of nitric oxide synthase, is regulated by the enzymatic participants of synthetic and metabolic processes, i.e., type I protein *N*-arginine methyltransferase (PRMT) and dimethylarginine dimethylaminohydrolase (DDAH). Previous reports have demonstrated that circulating ADMA levels can vary in patients with type 1 and type 2 diabetes mellitus (T2DM). White adipose tissue expresses the full enzymatic machinery necessary for ADMA production and metabolism; however, modulation of the activities of adipose ADMA-related enzymes in T2DM remains to be determined.

**Methods:**

A rodent model of T2DM using 11- and 20-week old Goto-Kakizaki (GK) rats was used. The expression and catalytic activity of PRMT1 and DDAH1 and 2 in the white adipose tissues (periepididymal, visceral and subcutaneous fats) and femur skeletal muscle tissue were determined by immunoblotting, *in vitro* methyltransferase and *in vitro* citrulline assays.

**Results:**

Non-obese diabetic GK rats showed low expression and activity of adipose PRMT1 compared to age-matched Wistar controls. Adipose tissues from the periepididymal, visceral and subcutaneous fats of GK rats had high DDAH1 expression and total DDAH activity, whereas the DDAH2 expression was lowered below the control value. This dynamic of ADMA-related enzymes in white adipose tissues was distinct from that of skeletal muscle tissue. GK rats had lower levels of serum non-esterified fatty acids (NEFA) and triglycerides (TG) than the control rats. In all subjects the adipose PRMT1 and DDAH activities were statistically correlated with the levels of serum NEFA and TG.

**Conclusion:**

Activities of PRMT1 and DDAH in white adipose tissues were altered in diabetic GK rats in an organ-specific manner, which was reflected in the serum levels of NEFA and TG. Changes in adipose ADMA-related enzymes might play a part in the function of white adipose tissue.

## Introduction

Asymmetric *N*^G^*N*^G^-dimethylarginine (ADMA), a naturally occurring inhibitor of nitric oxide synthase (NOS), is produced by the proteolysis of intracellular proteins that are posttranslationally modified by type I protein *N*-arginine methyltransferase (PRMT) [1]. PRMT1 is a dominant type I enzyme responsible for generating over the 85% of total *N*-methyltransferase activity in mammalian cells [1]. Dimethylarginine dimethylaminohydrolase 1 and 2 (DDAH1/2) catalyze the hydrolysis of ADMA into L-citrulline and dimethylamine [2]. Therefore, PRMT1 and DDAH1/2 can determine the bioavailable cellular ADMA levels and subsequent local NO levels [3]. Human adipocytes and white adipose tissue have been shown to express the full enzymatic machinery required for ADMA production and metabolism [4].

The Goto-Kakizaki (GK) rat, an inbred congenic strain with type 2 diabetes mellitus (T2DM) without apparent obesity [5], is not known to have any major defects in the production and/or the receptor expression of adipocytokines [6]. Neither the macrophage density nor macrophages-per-adipocyte ratio is altered in GK rats; however, the blood flow in their white adipose tissue is markedly increased with the considerable contribution of the NOS/NO system [7,8].

Based upon these characteristics of GK rats, the present study aims to explore the possible involvement of PRMT1 and DDAH1/2 in the function of white adipose tissue through the dynamics and characteristic of these ADMA-related enzymes in T2DM.

## Methods

### Animals

Both 11- and 20-week-old male Wistar and GK rats were housed with free access to water and standard rat diet (352 kcal/100 g) and measured for body-weight. The animal care and all experimental protocols followed the National Institutes of Health guidelines and were approved the local Ethics Committee. The GK rats did not show any gross change in their body-weight or liner growth compared with Wistar controls. Male GK rats as well as Wistar rats have shown to reach sexual maturity at approximately 10–12 weeks of age, suggesting that impaired sexual maturation is unlikely to occur in the GK rats.

### Sample preparation

Blood samples were harvested from the tail vein of the rats during the postprandial period. Serum non-esterified fatty acids (NEFA) and triglycerides (TG) levels were determined by standard enzymatic methods (Wako Pure Chemical, Tokyo, Japan). After the rats were sacrificed, periepididymal, visceral and subcutaneous fats and femur muscle tissues were immediately harvested. Tissue extracts were then prepared by the dissection and homogenization of the tissues in ice-cold lysis buffer [25 mM HEPES, pH 7.9, 1% NP-40, 137 mM NaCl, 1 × protease inhibitor cocktail (Roche, Indianapolis, IN)] using a Polytron homogenizer (Brinkman Instruments, Westbury, NY) at 4°C. The samples were centrifuged at 2000 × *g* for 5 min at 4°C, and the resulting supernatants were then recentrifuged at 14000 × *g* for 20 min at 4°C. The aliquots were used in the following experiments.

### Immunoblotting

Immunoblot experiments were performed as previously described [9]. The tissue extracts were resolved using 3 × SDS-polyacrylamide gel electrophoresis (PAGE) buffer (187.5 mM Tris–HCl, 6% SDS, 30% glycerol, 150 mM dithiothreitol, 0.3% bromophenol blue, pH 6.8), and the samples were boiled for 5 min at 95°C. After centrifugation at 15000 × *g*, the aliquots were subjected to 7.5% SDS-PAGE. The proteins in the gel were transferred to a nitrocellulose membrane (GE Healthcare Biosciences, Tokyo, Japan) by electroblotting. The membranes were treated with anti-PRMT1 (1:4000, Upstate Biotechnology, Lake Placid, NY), anti-DDAH1 (1:1000, Santa Cruz Biotechnology, Santa Cruz, CA), anti-DDAH2 (1:2000, Santa Cruz Biotechnology), or anti-actin antibody (1:5000, Sigma-Aldrich, St. Louis, MO) followed by appropriate secondary antibody conjugated to HRP (1:10000–20000, Santa Cruz Biotechnology and GE Healthcare Biosciences). Immunoreactive proteins were detected using the ECL Plus system (GE Healthcare Biosciences). For the quantification of the results, the bands were measured using the NIH Image software (Image J version 1.2; National Institutes of Health, Bethesda, MD).

### In vitro methyltransferase assay

The tissue extracts (1 mg) were subjected to immunoprecipitation with a monoclonal anti-PRMT1 antibody (Abcam, Cambridge, MA) bound to protein A/G agarose (Santa Cruz Biotechnology). *In vitro* methylation was achieved by adding the immune complexes to R3 peptide (Ac-GGRGGFGGRGGFGGRGGFG-NH_2_; Invitrogen, Carlsbad, CA) and ^3^H]-*S*-adenosylmethionine (AdoMet) (148 kBq; GE Healthcare Biosciences) in a final volume of 35 μl with PBS for 90 min at 37°C as previously described [9]. The reaction was terminated by adding 20 μl 3 × SDS-PAGE buffer. The samples were heated at 95°C for 5 min, chilled at 4°C for 5 min, and centrifuged at 15000 × *g*. The supernatants were subjected to SDS-PAGE in a 5% Tris–HCl gel, transferred to a poly(vinylidene difluoride) membrane (Millipore, Billerica, MA), sprayed with En^3^hance (PerkinElmer Life and Analytical Sciences, Boston, MA), and exposed to Kodak BioMax MS film (Eastman Kodak Company, Rochester, NY) with the Transcreen LE Intensifying Screen (Eastman Kodak Company) for 2–5 days at −80°C. Following exposure, the membrane was washed twice with the transfer buffer and then stained with Coomassie brilliant blue for 5 min to detect the amount of R3 peptides transferred to the membrane. The methyltransferase activity was quantified by measuring the signal on the exposed blot using Image-J.

### In vitro citrulline assay

DDAH activity was assessed by an L-citrulline assay adapted from a previous report [10]. Tissue extracts (300 μg) were incubated with urease (100 U/ml; Sigma-Aldrich) for 15 min at 37°C, and then 400 μl of ADMA (1 mM; Alexis Biochemicals, Plymouth Metting, PA) in sodium phosphate buffer was added to 100 μl of each sample for 45 min at 37°C. The reaction was terminated by the addition of 500 μl of 4% sulfosalicylic acid, and the samples were mixed and centrifuged at 3000 × *g* for 10 min. The supernatant (100 μl) and L-citrulline standard (3.125—100 μM; Alexis Biochemicals) were then divided into a 96-well ELISA plate (Nalge Nunc International, Tokyo, Japan) in triplicate, and 100 μl of the color mixture (33% v/v of oxime reagent with 66% v/v of antipyrine/H_2_SO_4_ reagent; Sigma-Aldrich) was added to each well. The plate was covered with a sealing film (EXCEL Scientific, Victorville, CA), shaken for 1 min, incubated in the dark for 110 min at 60°C, and then cooled in an ice bath for 10 min. The activity was then measured by spectrophotometric analysis at 450 nm/620 nm (Immuno Mini NJ-2300, Nihon InterMed, Tokyo, Japan).

### Statistical analysis

Statistical analyses were performed using the StatView software (SAS Institute Inc., Cary, NC). Data are presented as the mean ± standard error of the mean (SEM). The comparisons between experimental groups were performed using an unpaired Student’s *t* test. The Pearson’s correlation coefficient was used for correlations. A *p* ≪ 0.05 was considered statistically significant.

## Results

**Figure 1:**
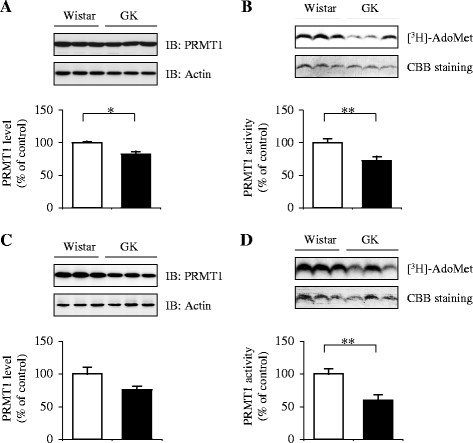
**Relative expression and enzymatic activity of PRMT1 in the white adipose tissue of GK rats.** The extracts of the periepididymal fat pads from 11- (**A**, **B**) and 20-week-old (**C**, **D**) Wistar (*open bars*) and GK rats (*closed bars*) were analyzed for PRMT1 expression and activity. Representative results are shown in the upper panels. Each bar in the graph represents the mean ± SEM (n = 5–10). The percentile represents the relative level of expression or activity to that of the age-matched Wistar controls. *p ≪ 0.05, **p ≪ 0.01 vs. the controls. *IB*, immunoblotting; *Ado-Met*, S-adenosylmethionine.

First, to examine the alteration of PRMT1 and DDAH1/2 expression and activity in the white adipose tissue of GK rats, the relative expression and activity were assessed using tissue extracts in the periepididymal fat. The PRMT1 protein level of periepididymal fat decreased by 12% in 11-week-old GK rats compared to that of the age-matched Wistar controls (*p* = 0.037) (Figure 1A). Consistent with the alteration of PRMT1 expression, the catalytic activity of PRMT1 in the tissue of 11-week-old GK rats was 28% lower compared to the corresponding control value (*p* = 0.003) (Figure 1B). Similar results were obtained from the adipose tissue of 20-week-old GK rats that had 23% less PRMT1 protein (*p* = 0.208) (Figure 1C) accompanied by a 40% reduction in the PRMT1 activity (*p* = 0.005) ( 1D) compared to the age-matched Wistar controls.

**Figure 2:**
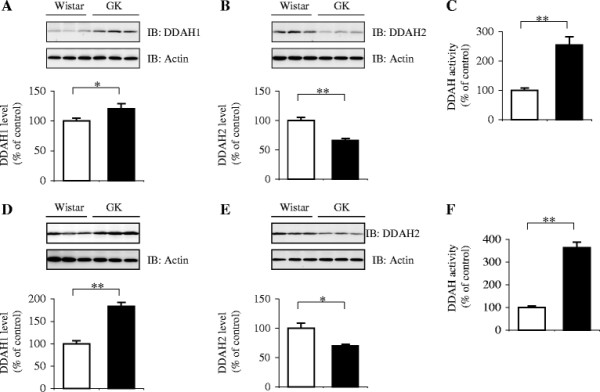
**Relative expression and enzymatic activity of DDAHs in the white adipose tissue of GK rats.** The extracts of the periepididymal fat pads from 11- (**A**–**C**) and 20-week-old (**D**–**F**) Wistar (*open bars*) and GK rats (*closed bars*) were analyzed for DDAH1 and DDAH2 expression, and total DDAH activity. Representative results are shown in the upper panels. Each bar in the graph represents the mean ± SEM (n = 5–10). The percentile represents the relative level of expression or activity to that of the age-matched Wistar controls. *p ≪ 0.05, **p ≪ 0.01 vs. the controls. *IB*, immunoblotting.

The DDAH1 protein level in the tissue extracts from the periepididymal fat of GK rats was increased by 20% at 11 weeks of age and 84% at 20 weeks of age compared with the age-matched controls (*p* = 0.046 and 0.003, respectively) (Figure 2A and D). In contrast, the DDAH2 protein levels were lower in the tissue of GK rats than in the control rats, with a 34% reduction for 11-week-old and a 30% reduction for 20-week-old rats compared to the corresponding control values (*p* ≪ 0.001 and *p* = 0.024, respectively) (Figure 2B and E). The catalytic activities of adipose DDAH in GK rats were 2.5-fold and 3.6-fold greater than the corresponding control values for 11- and 20-week-old rats, respectively (*p* ≪ 0.001 and *p* ≪ 0.001, respectively) (Figure 2C and F).

To examine whether the above-mentioned modification applies to the other localized adipose tissues or a different organ, the expression and activities of PRMT1 and DDAH1/2 were evaluated in visceral and subcutaneous fats and skeletal muscle tissue (Table 1). The patterns of modification in the extracts of visceral and subcutaneous fats from 11-week-old GK rats were nearly identical with those of periepididymal fat, showing significant decreases in PRMT1 and DDAH2 expression and PRMT1 activity and increases in DDAH1 expression and DDAH activity compared to the age-matched Wistar controls. However, the characteristics of ADMA-related enzymes in the skeletal muscle tissues of 11- and 20-week-old GK rats were distinct from those of the white adipose tissues, suggesting that white adipose tissues of GK rats might express and regulate for ADMA-related enzymes in an organ-specific manner.

**Table 1:** Expression and activities of ADMA-related enzymes in the white adipose and skeletal muscle tissues of control Wistar and diabetic GK rats

	**PRMT1 expression**		**PRMT1 activity**			
	**Wistar**	**GK**	**Wistar**	**GK**		
Visceral fat	100 ± 2	75.7 ± 6.1**	100 ± 3	41.9 ± 6.4**		
Subcutaneous fat	100 ± 4	79.0 ± 3.3**	100 ± 5	73.4 ± 6.4**		
Skeletal muscle (11 weeks of age)	100 ± 4	109 ± 9	100 ± 4	102 ± 3		
Skeletal muscle (20 weeks of age)	100 ± 2	117 ± 2	100 ± 3	125 ± 4**		
	**DDAH1 expression**		**DDAH2 expression**		**DDAH activity**	
	**Wistar**	**GK**	**Wistar**	**GK**	**Wistar**	**GK**
Visceral fat	100 ± 9	138 ± 9	100 ± 7	68.7 ± 6.5**	100 ± 7	259 ± 19**
Subcutaneous fat	100 ± 10	139 ± 5*	100 ± 5	73.5 ± 3.3*	100 ± 8	245 ± 33*
Skeletal muscle (11 weeks of age)	100 ± 10	90.8 ± 2.3	100 ± 21	136 ± 11	100 ± 9	91.4 ± 9.3
Skeletal muscle (20 weeks of age)	100 ± 5	96.0 ± 5.4	100 ± 5	144 ± 7**	100 ± 5	109 ± 6

Values represent the mean ± SEM of five to ten rats in each group and are expressed as the percentile for the relative level of expression or activity to that of the age-matched Wistar controls. *p ≪ 0.05, **p ≪ 0.01 vs. the controls.

**Figure 3:**
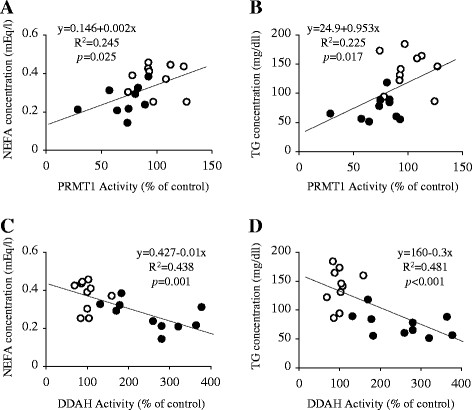
**Correlation analysis of the relationships between the activities of ADMA-related enzymes and lipid biomarker levels.** The values of the parameters are depicted for 11-week-old Wistar (*open circles*) and GK rats (*closed circles*) in Figure 1B, Figure 2C and Table 2. The lines show linear regressions for the parameters indicated in each graph. The Pearson’s correlation coefficients (R^2^) and *p* values for each correlation are also indicated in the graph.

Characteristics of Wistar control and GK diabetic rats at 11 and 20 weeks of age are shown in Table 2. Both 11- and 20-week-old GK rats were equivalent to the age-matched Wistar controls in body-weight (*p* = 0.408 and *p* = 0.496, respectively). During the postprandial state, the serum levels of NEFA were lower in 11- and 20-week-old GK rats compared to the age-matched Wistar controls (*p = *0.027 and *p* = 0.002, respectively). GK rats also exhibited lower TG levels compared to the corresponding control values (*p* ≪ 0.001 for 11-week-old and *p* ≪ 0.001 for 20-week-old). For all 11-week-old subjects, a significant positive linear correlation was observed between PRMT1 activity and serum NEFA or TG level (*p* = 0.025 and *p* = 0.017, respectively) (Figure 3A and B). A negative correlation was also observed between DDAH activity and the serum NEFA or TG level (*p* = 0.001 and *p* ≪ 0.001, respectively) (Figure 3C and D).

**Table 2:** Characteristics of Wistar control and GK diabetic rats at 11 and 20 weeks of age

	**11 weeks of age**		**20 weeks of age**	
	**Wistar**	**GK**	**Wistar**	**GK**
Body-weight (g)	250 ± 4	242 ± 5	329 ± 5	323 ± 6
Non-esterified fatty acids (mEq/l)	0.38 ± 0.02	0.26 ± 0.02*	0.43 ± 0.01	0.22 ± 0.03**
Triglycerides (mg/dl)	140 ± 10	76 ± 6**	159 ± 14	88 ± 7**

Values represent the mean ± SEM of five to ten rats in each group. *p ≪ 0.05, **p ≪ 0.01 vs. the age-matched Wistar control rats.

## Discussion

The present study demonstrated that the white adipose tissue of diabetic GK rats exhibited a high level of DDAH1 expression accompanied by enhanced DDAH activity compared to controls, whereas PRMT1 expression and activity were reduced in the tissue. The dynamics of adipose ADMA-related enzymes is apparently distinct from those of skeletal muscle tissues, indicating that this modification is likely to occur in an organ-specific manner. The adipose ADMA-related enzyme activities reflected serum levels of lipid biomarkers such as NEFA and TG. These data suggest that the adipose ADMA metabolism may be flexibly modulated in metabolic disorders, and may be involved in the function of white adipose tissue.

It has been reported that liver and pancreas lysates from GK rats displayed low PRMT1 activities and this may be associated with defective hepatic insulin signaling, excessive gluconeogenesis in the liver, and inappropriate glucose-stimulated insulin secretion in GK rats [9]. The present study demonstrates that the white adipose tissue of GK rats had lower PRMT1 activity relative to control Wistar rats. Because the tissue lysates of GK rats exhibited a low degree of PRMT1 expression, the decreased activity may be partly responsible for the low protein level. The present study also revealed that GK rats exhibit an increase in adipose DDAH1 expression, whereas adipose DDAH2 expression was diminished compared to the controls. The catalytic activity of adipose DDAH in GK rats was greater than that of the controls. Adipose DDAH1 expression is concomitant with DDAH activity and is, therefore consistent with the previous report on human cultured adipocytes and white adipose tissue [4]; DDAH1 may predominate in this tissue of the subjects.

The mechanism(s) for the modification of PRMT1 and DDAH1/2 expression in the adipose tissue of GK rats remains to be determined. Interestingly, an opposite pattern of the expression of the DDAH subtypes is proposed in the kidneys of streptozotocin-induced diabetic rats. DDAH1 expression is decreased but PRMT1 and DDAH2 expression is increased under angiotensin II (AngII) type 1 receptor (AT_1_-R) activation [11]. Furthermore, AT_1_-R activation in the rat retina also leads to high PRMT1 and low DDAH1 expression, which is implicated in the progression of diabetic retinopathy [12]. An increase in circulating NEFA levels by lipid/heparin infusion mimics the abnormal lipid profile in patients with insulin resistance and causes endothelial dysfunction via an AT_1_-R-dependent reduction of NO synthesis [13]. In agreement with these reports, incubation of differentiated 3T3-L1 adipocytes with either AngII or saturated fatty acid (palmitic acid) results in increased PRMT1 expression and decreased DDAH1 expression (Iwasaki, unpublished observation). Taken together, NEFA could suppress the production of bioavailable adipose NO subsequent to the modulation of local ADMA-related enzymes through the AT_1_-R-dependent pathway, which may partly account for the relationships between lipid biomarker levels and adipose PRMT1 or DDAH activity.

The IC_50_ of ADMA on NOS is approximately 10 μM and the intracellular ADMA levels are normally well above this IC_50_ level due to intracellular accumulation via the cationic amino acid transporter [2,3]. A 50% increase in cellular DDAH activity by DDAH1 overexpression results in a 22% increase in intracellular NO production [14], indicating that the change in the adipose ADMA-related enzyme activities in GK rats may be sufficient to enhance local NO production. GK rats have increased blood flow in white adipose tissue [7,8]. Although a major proportion of the postprandial enhancement appears to be under β-adrenergic control, the NOS/NO system contributes considerably to hyperperfusion in this tissue [7,8]. Taken together, the modification of adipose ADMA-related enzyme activities may be implicated in the enhanced white adipose tissue blood flow in GK rats and possibly associated with the machinery for adipose functions.

## Competing interests

The author declares that there are no competing interests.

## Author’s contributions

HI conceived and designed the study, performed the experiments, and wrote the manuscript.
